# No Differences in Gastrointestinal Bleeding Risk among Clopidogrel-, Ticagrelor-, or Prasugrel-Based Dual Antiplatelet Therapy

**DOI:** 10.3390/jcm9051526

**Published:** 2020-05-18

**Authors:** Viviana Laredo, Carlos Sostres, Sandra García, Patricia Carrera-Lasfuentes, Pablo Revilla-Marti, Ángel Lanas

**Affiliations:** 1 Department of Gastroenterology, University Clinic Hospital Lozano Blesa, 50009 Zaragoza, Spain; sgarciamateo7@gmail.com (S.G.); alanas@unizar.es (Á.L.); 2 CIBERehd, 28029 Madrid, Spain; pcarreralasfuentes@gmail.com; 3 IIS Aragón, 50009 Zaragoza, Spain; 4 Department of Cardiology, University Clinic Hospital Lozano Blesa, 50009 Zaragoza, Spain; pablorevillamarti@gmail.com; 5 Department of Medicine, Psychiatry and Dermatology, University of Zaragoza, 50009 Zaragoza, Spain

**Keywords:** ticagrelor, prasugrel, gastrointestinal bleeding, dual antiplatelet therapy

## Abstract

The risk for gastrointestinal bleeding from dual antiplatelet therapy (DAPT) with new antiplatelets (prasugrel/ticagrelor) compared to clopidogrel is unclear. Aim: To determine the risk and type of major (gastrointestinal bleeding requiring hospitalization) and minor (anemia and iron deficiency) gastrointestinal events with different types of DAPT. Methods: Retrospective observational cohort study of patients who started DAPT after percutaneous coronary intervention. Follow-up was censored after 12 months of DAPT, when a major gastrointestinal event occurred, or when DAPT was discontinued. Results: Among 1,327 patients (54.03% were treated with clopidogrel-based DAPT, 38.13% with ticagrelor-based DAPT, and 7.84% with prasugrel-based DAPT), 29.5% had at least one gastrointestinal event. Patients taking clopidogrel-DAPT were older, with more comorbidities, and higher gastrointestinal risk compared to those taking other DAPT regimens. Adjusted hazard ratios (HRs) showed no between-group differences in the risk for major (clopidogrel vs. new antiplatelets: HR 0.996; 95% confidence interval 0.497–1.996) and minor (HR 0.920; 0.712–1.189) gastrointestinal events. Most patients received proton pump inhibitors while on DAPT (93.3%) and after withdrawal (83.2%). Conclusion: Prasugrel- or ticagrelor-based DAPT was not associated with increased gastrointestinal bleeding risk when compared to clopidogrel-DAPT. New antiplatelets do not necessarily need to be restricted to patients with low gastrointestinal risk.

## 1. Introduction

Dual antiplatelet therapy (DAPT) is more effective than aspirin (ASA) alone in the treatment of patients after acute coronary syndrome [1]. Classically, DAPT was based on ASA and clopidogrel, which is a thienopyridine prodrug that inhibits the P2Y_12_ receptor and decreases major cardiovascular adverse events [2]. However, there is wide variability in its effect due to its pharmacokinetic characteristics [3]. For that reason, two new inhibitors of the P2Y_12_ receptor (prasugrel and ticagrelor) that have demonstrated a faster and more effective platelet inhibition as compared with clopidogrel [4] are being used now.

The risk for gastrointestinal bleeding associated with clopidogrel has been well established, especially in elderly patients and in combination with ASA and nonsteroidal anti-inflammatory drugs (NSAIDs) [5]. Clopidogrel’s antiplatelet effect increases the risk of bleeding from pre-existing mucosal lesions [6,7]. Patients taking aspirin or clopidogrel may develop both upper and lower gastrointestinal bleeding, and the risk of lower gastrointestinal bleeding in ASA or NSAID users can be higher likely because of the use of proton pump inhibitors (PPIs) [8]. The risk of gastrointestinal bleeding in patients treated with prasugrel or ticagrelor DAPT is not well defined.

Based on the Thrombolysis in Myocardial Infarction (TIMI) definition of bleeding, patients treated with prasugrel had increased bleeding risk compared with those taking clopidogrel in the *Trial to assess improvement in therapeutics outcomes by optimizing platelet inhibition with prasugrel* (TRITON trial) [9]. Similarly, in the *Study of platelet inhibition and patient outcomes* (PLATO trial) [10], compared with the clopidogrel group, the ticagrelor group had a higher risk of major bleeding that was not related to coronary artery bypass graft. However, subsequent studies have yielded contradictory results, with an increased risk of bleeding with prasugrel and ticagrelor in some of them [9,10,11,12,13,14,15], but no differences between clopidogrel and these new compounds in others [16,17,18,19,20,21,22]. The main limitation of acquiring meaningful comparisons among studies and these antiplatelet agents is the variability in bleeding definitions used [23,24]. When a standardized definition is used, the differences in bleeding incidence between studies diminishes [25]. Moreover, most studies have used global bleeding as an endpoint and not specifically gastrointestinal bleeding, which is the most frequent event [15].

Despite this mixed evidence, the potential higher risk of bleeding associated with new antiplatelet agents has been translated into clinical practice, so that their use has been limited to younger patients with fewer comorbidities [23,26,27,28]. The paradoxical outcome is that these new antiplatelets are not used in patients with higher cardiovascular risk and who might benefit the most. Additionally, the management of DAPT in patients with gastrointestinal bleeding is a challenge in clinical practice because recent evidence suggests a benefit from the early resumption of antiplatelet therapy [29]. Given this lack of conclusive findings, we conducted a study to determine the risk for specific types of major and minor gastrointestinal events in patients taking DAPT, depending on the type of treatment used.

## 2. Material and Methods

This retrospective, observational cohort study included patients from two general hospitals in Spain who started DAPT after a percutaneous coronary intervention (PCI) between 1 January 2015, and 31 December 2016. Patients treated with DAPT at the time of the PCI or who discontinued DAPT during the first month of treatment were excluded. The study flowchart and the STROBE checklist from observational studies [30] are available in the supplementary material (Figure S1 and Table S1, respectively).

The follow-up period was censored after 12 months of treatment, when a major gastrointestinal event occurred, when DAPT was definitively discontinued, or at death. Demographics, data from the cardiovascular event, comorbidities, previous gastrointestinal events, concomitant treatment (e.g., NSAIDs, anticoagulants, PPI, steroids), and events during follow-up were obtained from the electronic clinical history. The events of interest during the follow-up were gastrointestinal events (major and minor), non-gastrointestinal bleeding events, cardiovascular events, and death. The primary endpoint was the occurrence of any gastrointestinal event of interest during the follow-up period, classified as major or minor. A major gastrointestinal event was defined as follows: any gastrointestinal bleeding leading to hospitalization or attention in the emergency room and consisting of the presence of hematemesis, melena, red blood per rectum, or an acute hemoglobin drop of more than 2 g/dL without evidence of visible gastrointestinal bleeding, when assessed by medical staff, and with no other non-gastrointestinal sources of bleeding. The bleeding was classified as upper or lower, depending on the location (proximal or distal to the ligament of Treitz). Bleeding without any identified source was classified as being obscure in origin.

A minor gastrointestinal event was defined as the development of anemia (hemoglobin <12 g/dL in women and <13 g/dL in men) associated with iron deficiency, or iron deficiency without anemia (serum ferritin < 30 ng/mL and transferrin saturation < 19%).

The gastrointestinal risk was defined according to the patient’s previous history of gastrointestinal bleeding or the presence of lesions that could increase the risk of bleeding (e.g., peptic ulcer, angiodysplasia, diverticula). Information concerning other ischemic events, non-gastrointestinal bleeding events, death, and drug management during a gastrointestinal event were also collected. Finally, we analyzed the use of concomitant treatment with PPI during and three months after DAPT withdrawal (at month 15).

### Statistical Analysis

We conducted an initial exploratory analysis of all variables included in the study. Qualitative variables are expressed as frequencies and percentages, whereas quantitative variables are reported as means with standard deviations (SDs). We performed between-group comparisons for qualitative variables using Chi-squared or Fisher’s exact tests. Means between two independent groups were compared with the Mann–Whitney U test, and we used the Kolmogorov–Smirnov test to assess the normal distribution of the variables. To determine the risk for both major and minor gastrointestinal events with new antiplatelets vs. clopidogrel, we applied univariate and multivariate Cox proportional hazards models with adjustment for age, sex, Charlson comorbidity index, anticoagulant treatment, PPI therapy, and previous gastrointestinal risk. Hazard ratios (HRs) with 95% confidence intervals (CIs) were used to estimate the strength of the association. For all tests, a two-sided p < 0.05 was considered statistically significant. All statistical analyses were performed using SPSS 26.0 for Windows (SPSS Ibérica, Madrid, Spain).

## 3. Results

### 3.1. Baseline Characteristics

A total of 1327 patients were included in the study: 54.03% (717/1327) started treatment with ASA and clopidogrel, 38.13% (506/1327) with ASA, and ticagrelor, and 7.84% (104/1327) with ASA and prasugrel. Baseline characteristics differed significantly between patients treated with ASA and clopidogrel and those treated with ASA and ticagrelor or prasugrel, as shown in Table 1.

### 3.2. Gastrointestinal Events During Follow-up

During the follow-up period, the incidence of the primary endpoint was more frequent in the group taking clopidogrel-DAPT than in the group taking new antiplatelets-DAPT (35.1% vs. 23.0%; *p* < 0.001). Major gastrointestinal bleeding events were similar between both groups of DAPT (3.5% clopidogrel-DAPT vs. 3.3% new antiplatelets; *p* = 0.880), but the frequency of minor gastrointestinal events (anemia and iron deficiency) was higher in the group taking ASA and clopidogrel compared to those taking ASA and new antiplatelets (31.7% vs. 19.7%; *p* < 0.001). However, after the adjustment for main confounding factors (age, sex, Charlson index, anticoagulant treatment, PPI therapy, previous gastrointestinal risk), there were no differences between groups in the risk of gastrointestinal events (Table 2).

A total of 45 patients had a major gastrointestinal event. Most events (30/45, 66.67%) were lower gastrointestinal bleeding, 26.67% (12/45) were of upper gastrointestinal origin, and 6.67% (3/45) were of obscure gastrointestinal origin. The causes of bleeding events are described in Table 3. 15.6% (7/45) of patients with gastrointestinal bleeding were on anticoagulant treatment (25% [3/12] of patients with upper GI bleeding and 13.3% [4/30] of patients with lower bleeding) and all of them were also taking clopidogrel-DAPT. Almost half of the patients with gastrointestinal bleeding received a blood transfusion (21/45), and 60% of them (27/45) were treated with intravenous iron, without differences between treatment (blood transfusion: 48% clopidogrel vs. 45% new antiplatelet, *p* = 1.00; intravenous iron: 68% clopidogrel vs. 50% new antiplatelet, *p* = 0.241). A total of 347 (26.1%) patients had a minor gastrointestinal event during the follow-up period, distributed as shown previously in Table 2.

### 3.3. Global Events During Follow-up

The incidence of global events of interest was also more common with clopidogrel-DAPT (45.0%) vs. new antiplatelet-DAPT (33.9%; *p* < 0.001). As shown in Table 4, the incidence of the overall gastrointestinal and non-gastrointestinal bleeding events did not differ, but the clopidogrel-DAPT group had more minor gastrointestinal events, cardiovascular events, and death. Among those with non-gastrointestinal bleeding events (n = 114), there were more patients also treated with anticoagulants in the group of clopidogrel-DAPT (15/56; 26.8%) than in the group of new antiplatelets-DAPT (3/58; 5.2%); *p* = 0.002. Information about the type of non-gastrointestinal bleeding and ischemic events is available in Appendix A, Table A1 and Table A2, respectively.

### 3.4. PPI Therapy and DAPT after a Major Gastrointestinal Event

After starting treatment with DAPT, 93.3% of patients were treated with PPI. Among those who developed gastrointestinal bleeding, at the time of the event, 86.7% were already on PPI treatment (91.7% of patients with upper gastrointestinal bleeding, 83.3% with lower, and 100% with an obscure origin, without differences between upper and lower; *p* = 0.655).

**Figure 1:**
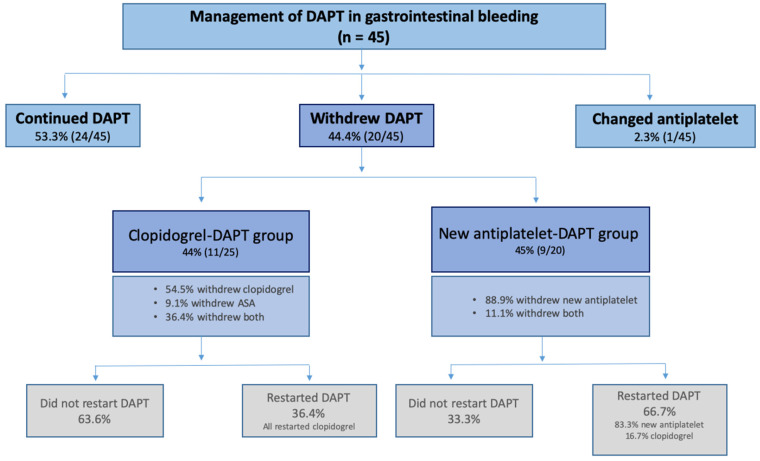
Management of DAPT in patients with gastrointestinal bleeding.

Among the patients with major gastrointestinal bleeding, DAPT was continued in 53.3% during and after the bleeding event. In 44.4% of these cases, DAPT was withdrawn, without differences between treatment groups (44% [11/25] clopidogrel vs. 45% [9/20] new antiplatelets; *p* = 1.000), as shown in Figure 1.

In both groups, only one antiplatelet was withdrawn from most patients (63.6% in the clopidogrel group and 88.9% in the new antiplatelets group). After the gastrointestinal event, 63.6% (7/11) of patients who withdrew clopidogrel-DAPT, continued therapy with one antiplatelet, whereas among those in the new antiplatelet-DAPT group, most patients (66.7%) restarted DAPT.

In the 15th month, 54.3% (721/1327) of the patients included in the study withdrew from DAPT. Most patients in that group continued treatment with PPI (83.2%; 600/721).

### 3.5. Causes of Death

During follow-up, 23 patients died (1.7%), almost half of them because of an ischemic event (11/23; 47.8%), 43.5% (10/23) because of non-ischemic and non-bleeding events, and 8.7% (2/23) because of a bleeding event. Only one patient died as a result of gastrointestinal bleeding.

## 4. Discussion

In this study, as previously described [23,26,27,28], we have confirmed that in clinical practice, the baseline characteristics of patients taking clopidogrel-DAPT are significantly different from those in treatment with new antiplatelet-DAPT. In European registries [23], patients treated with prasugrel are usually younger than those treated with ticagrelor [31], and patients treated with clopidogrel are older, with almost 10 years of difference in some studies. Danchin et al. [23] showed that older age is usually associated with more comorbidities and higher cardiovascular risk, as we confirm in our study. Paradoxically, patients with a worse cardiovascular profile are being treated with clopidogrel [23,26,27], which can be less effective than the new antiplatelets [9,10]. In an Israeli study, age over 75 years (odds ratio [OR] 0,17; 95% CI 0.10–0.27), non-ST segment elevation myocardial infarction (OR 0.37; 95% CI 0.26–0.54), and prior cardiovascular event or stroke (OR 0.41; 95% CI 0.2–0.68) were factors associated with under treatment with new antiplatelets [26].

In addition to the most common baseline characteristics reported in previous studies, we have analyzed the effect of previous gastrointestinal diseases in both groups of treatment and the use of concomitant anticoagulant therapy. We found that there were more patients with these characteristics in the clopidogrel-DAPT group, indicating that these patients may be undertreated with the new antiplatelets.

The superior efficacy of DAPT based on new antiplatelet compounds has been well established [9,10], but data are lacking on the risk and type of gastrointestinal bleeding with the new antiplatelet-based DAPT. In cardiology studies, bleeding is the most common non-ischemic complication in patients with acute coronary syndromes and has been associated with poor prognosis. Moreover, the gut is the most common location of bleeding events [24,32]. Our results support the importance of gastrointestinal events because more than one in four patients treated with DAPT (29.5%) had at least one gastrointestinal event. The most common gastrointestinal event was anemia, which may have a negative effect on cardiovascular disease and has not been analyzed in previous reports [15].

According to the type of therapy, the risk of gastrointestinal bleeding did not differ between clopidogrel-based DAPT and new antiplatelet-based DAPT group. These results support the conclusions of some studies [16,17,18,19,20,21,22] but differ from others [9,10,11,12,13,14,15]; however, the designs are not comparable. First, most studies have analyzed bleeding as a secondary endpoint and determined the overall risk of bleeding (including, e.g., epistaxis, intracranial, and hematuria) and not specifically gastrointestinal events. European registries contain detailed information about ischemic events, but few include data concerning bleeding in general; if they do so, the focus is more on severity than the cause and location [25]. In fact, most registries report only data about major bleeding events. Furthermore, there is a wide variability in bleeding definitions among studies (e.g., using the Bleeding Academic Research Consortium [BARC] vs. CRUSADE vs. TIMI criteria) [24], which complicates comparisons, as a recent meta-analysis found [15]. In addition to the definitions, most studies report bleeding events only during hospitalization, whereas the timing of the bleeding ranges from 15 days to 15 months [25]. Quinlan et al. analyzed the incidence of bleeding in the European registries using the TIMI criteria compared to the study criteria (CURE, PLATO, CURRENT). They found a lower incidence of bleeding and no differences between clopidogrel-based DAPT and new antiplatelets-based DAPT [25]. The BARC developed a new definition of bleeding to standardize the information from the studies [24]; however, it is still a definition that includes overall bleeding, and not specifically gastrointestinal events, which makes comparison with our data difficult.

To our knowledge, the only study that provides information specifically about gastrointestinal bleeding is a meta-analysis using data from randomized controlled trials [15]. The authors concluded that new antiplatelets are associated with an increased risk for gastrointestinal bleeding. However, most of the information gathered comes from cardiology studies, with the limitations noted above. Authors suggest that data on bleeding are underreported and advocate for new studies that are more focused on the definition of bleeding-related complications.

Our study provides information about the location and the etiology of gastrointestinal bleeding. More patients had lower than upper gastrointestinal bleeding, in agreement with findings from our previous studies analyzing gastrointestinal bleeding events in patients taking DAPT [8] and antiplatelets in general [29]. In fact, currently, there are more hospital admissions of patients presenting with lower than with upper gastrointestinal bleeding [33], which can be explained in part by the large number of patients receiving concomitant treatment with PPI [8]. In our study, half of the cases of upper gastrointestinal bleeding could not be prevented with PPI because they were not peptic lesions.

Bleeding in patients with DAPT represents a challenge in clinical practice because doctors must carefully assess the balance between bleeding and ischemic risks. Our study provides information from real clinical practice. Fewer than half of the patients withdrew from DAPT and in most cases, only one antiplatelet was withdrawn, which agrees with current guideline recommendations [34]. After the event, more patients in the new antiplatelets group restarted DAPT when compared with clopidogrel-based DAPT. Finally, in contrast to other studies [35], our results indicate good adherence to the guidelines [36], which recommend PPI-concomitant treatment in patients on DAPT. On the other hand, after DAPT withdrawal, most patients continued PPI therapy even though many of them likely no longer showed any indication for the need for continued treatment. This pattern matches the current trend in some European countries of PPI overuse, in which once the treatment is prescribed, it continues long-term even when the indication has resolved [37].

Our study has limitations. Despite the large number of patients included in the follow-up, there were few cases of major gastrointestinal bleeding; however, the data do not show a trend between groups that might have suggested differences had sample sizes been larger. Another limitation is the difference between baseline characteristics of the groups, rendering comparisons between them difficult, but adjustment for confounders in part mitigated this issue. Nevertheless, it is possible that other residual confounders still bias the results. Finally, the retrospective nature of the study is a limitation. In this way, we could not provide the GI causes of anemia and iron deficiency. The strength of the study is the focus on the risk and type of gastrointestinal bleeding, given the current lack of studies addressing specifically this type of bleeding; most of them report on cardiovascular events or bleeding in general. Moreover, most bleeding events were fully investigated with endoscopic explorations and detailed information about the event characteristics, concomitant treatment, needed for transfusion, and management of DAPT. Finally, another strength is the report on anemia, an adverse event linked to the use of gastrotoxic drugs that have not been explored appropriately and may have a huge impact on outcome [32].

## 5. Conclusions

In summary, in this study, nearly one in three patients had a gastrointestinal event during the first year of DAPT, which is a frequent complication related to DAPT. This gastrointestinal risk was similar between clopidogrel and new antiplatelet DAPT after adjustment for confounding factors. Anemia and iron deficiency were the most common adverse events, which most studies have overlooked. Death during DAPT is related mainly to ischemic cardiovascular complications with low involvement of gastrointestinal bleeding, but the decision to prescribe clopidogrel vs. new antiplatelet compounds is based on bleeding risk. These results should be investigated in further studies to extend the indications for and cardiovascular benefits of new antiplatelets to less selected patient groups.

## Figures and Tables

**Table 1:** Baseline characteristics.

	Total (*N* = 1327)	ASA + Clopidogrel (*n* = 717)	ASA + New Antiplatelets (*n* = 610)	*p* Value
Sex (male), n (%)	1019 (76.8)	527 (73.5)	492 (80.7)	**0.002**
**Age (mean ± SD)**	66.9 ± 12.6	71.4 ± 11.6	61.5 ± 11.7	**<0.001**
**Charlson comorbidity index (mean ± SD)**	3.2 ± 2.5	4.2 ± 2.5	1.9 ± 1.9	**<0.001**
**Anticoagulant treatment**, n (%)	173 (13.0)	148 (20.6)	25 (4.1)	**<0.001**
PPI therapy, n (%)	1238 (93.3)	661 (92.2)	577 (94.6)	0.098
Previous GI risk, * n (%)	261 (19.7)	168 (23.4)	93 (15.2)	**<0.001**

GI: gastrointestinal. * History of gastrointestinal bleeding, diverticulosis, angiodysplasia, peptic ulcer, or gastrointestinal tumors.

**Table 2:** Adjusted risk for gastrointestinal (GI) events of dual antiplatelet therapy with new antiplatelets vs. clopidogrel.

	ASA + Clopidogrel (*n* = 717)	ASA + New Antiplatelets (*n* = 610)	HR (95% CI)	*p* Value	Adjusted HR (95% CI)	*p* Value
**Any GI event**, n (%)	252 (35.1)	140 (23.0)	0.572 (0.465–0.704)	**<0.001**	0.935 (0.735–1.188)	0.581
**Major GI event**, n (%)	25 (3.5)	20 (3.3)	0.877 (0.487–1.579)	0.662	0.996 (0.497–1.996)	0.991
**All minor GI events**, n (%)	227 (31.7)	120 (19.7)	0.546 (0.437–0.681)	**<0.001**	0.920 (0.712–1.189)	0.524
**Minor GI event, anemia**, n (%)	201 (28.0)	96 (15.7)	0.495 (0.388–0.632)	**<0.001**	0.876 (0.663–1.157)	0.351
**Minor GI event,iron deficiency**, n (%)	26 (3.6)	24 (3.9)	1.014 (0.582–1.767)	0.960	1.110 (0.581–2.124)	0.751

**Table 3:** Location and cause of major GI events.

Location	Cause	*n* (%)
**Upper**	Mallory–Weiss syndrome Gastric ulcer Gastric cancer Vascular gastric lesions Gastroduodenal erosions Vascular duodenal lesions Other	12 (26.67) 1 2 1 2 3 2 1
**Lower**	Hemorrhoidal bleeding Large bowel diverticulosis Ischemic colitis Colorectal cancer Angiodysplasia Other * Unknown	30 (66.67) 6 4 4 4 4 6 2
**Obscure**		3 (6.67%)

* four cases after polyp removal, one case after colonic biopsy, and one case with unidentifiable cause of macroscopic colonic bleeding.

**Table 4:** Events during follow-up by treatment group.

	Total (*N* = 1327)	ASA + Clopidogrel (*n* = 717)	ASA + New Antiplatelets (*n* = 610)	*p* Value
Major gastrointestinal event, *n* (%)	45 (3.4)	25 (3.5)	20 (3.3)	0.880
All minor gastrointestinal events, *n* (%)	347 (26.1)	227 (31.7)	120 (19.7)	**<0.001**
Non-gastrointestinal bleeding events, * *n* (%)	114 (8.6)	56 (7.8)	58 (9.5)	0.281
Ischemic or cardiovascular events, ** *n* (%)	131 (9.9)	82 (11.4)	49 (8.0)	**0.042**
Death	23 (1.7)	21 (2.9)	2 (0.3)	**<0.001**

* See Table A1 in Appendix A. ** See Table A2 in Appendix A.

## Supplementary Materials

The following are available online at https://www.mdpi.com/2077-0383/9/5/1526/s1, Figure S1: Study flowchart, Table S1: STROBE checklist.

## Appendix A

**Table A1:** Non-gastrointestinal bleeding events.

	Total *n* = 114	Clopidogrel-DAPT *n* = 56	New Antiplatelet-DAPT *n* = 58
**Epistaxis**, n (%)	38 (33.3)	19 (33.9)	19 (32.8)
**Hematuria**, n (%)	29 (25.4)	15 (26.8)	14 (24.1)
**Hematoma**, n (%)	25 (21.9)	12 (21.4)	13 (22.4)
**Intracranial bleeding**, n (%)	6 (5.3)	4 (7.1)	2 (3.4)
**Other**, n (%)	16 (14.0)	6 (10.7)	10 (17.2)

**Table A2:** Ischemic and other cardiovascular events.

	Total *n* = 131	Clopidogrel-DAPT *n* = 82	New Antiplatelet-DAPT *n* = 49
**Angina**, n (%)	71 (54.2)	44 (53.7)	27 (55.1)
**Non-ST elevation acute coronary syndrome**, n (%)	30 (22.9)	19 (23.2)	11 (22.4)
**ST elevation acute coronary syndrome**, n (%)	9 (6.9)	7 (8.5)	2 (4.1)
**Stroke**, n (%)	9 (6.9)	3 (3.7)	6 (12.2)
**Other, *** n (%)	12 (9.2)	9 (11)	3 (6.1)

* Other: peripheral embolism, pulmonary embolism.
